# A Case Report of a 16 Year Old With Catatonia With No Clear Medical or Psychiatric Cause

**DOI:** 10.1192/bjo.2024.655

**Published:** 2024-08-01

**Authors:** Andrew Breakwell, Claire Kelly

**Affiliations:** Beechcroft, Belfast, United Kingdom

## Abstract

**Aims:**

16 year old male with previous diagnoses of autism spectrum disorder and severe anxiety disorder was referred to Child and Adolescent Mental Health Services (CAMHS). His presentation included increasing anxiety, difficulty articulating his thoughts and emotions, difficulty completing tasks, school non-attendance, reduced food intake and possible auditory hallucinations. Risperidone was trialled in the community however refused after 5 days due to “brain fog”. He was seen by CAMHS community team twice weekly for 3 months prior to his emergency detained admission to adolescent psychiatric inpatient unit, due to no oral intake for 72 hours.

Family history included schizophrenia, bipolar disorder, depression and anxiety.

**Methods:**

Upon admission, symptoms observed included reduced verbal communication, psychomotor retardation, low mood, agitation, sleep difficulties, ambitendency, echolalia and poor oral intake. He had a Bush-Francis rating score of 8 and given a working diagnosis of catatonia. He responded positively to a lorazepam challenge therefore commenced on 1mg of lorazepam twice daily. Despite increasing doses, the catatonia worsened with severe psychomotor retardation, “psychological pillow” and nil food or fluid intake with Bush Francis score of 18. ECT was arranged as an emergency treatment but put on hold while tolerating all food and fluids requirements via nasogastric tube. Lorazepam dose was titrated to 3mg three times daily but signs seen of benzodiazepine toxicity therefore dose was reduced and ECT arranged for treatment resistance. Improvement seen on reduced dose prior to receiving ECT, therefore ECT put on hold again. His lorazepam dose was titrated up at a slower rate to 4mg three times daily which he was able to tolerate. His catatonia fully resolved at 12mg. Once stable, lorazepam dose was very gradually decreased until stopped. No evidence of catatonia returned.

**Results:**

Medical and psychiatric causes of catatonia were explored.

Two positive blood anti-NMDA receptor tests two months apart; both 1/10 titre. This was discussed with the specialist neurology team in Oxford who advised this was an incidental finding with no clinical implication (1% of healthy population are positive).

Throughout admission, possible fleeting psychotic and depressive symptoms were noted, including not trusting food, hallucinations, worries about contamination and apathetic mood. However, these all improved as the catatonia was treated.

**Conclusion:**

There was no clear underlying psychiatric or medical illness identified as a cause of the patient’s catatonia. Catatonia has a higher prevalence in people with autism. At discharge he was well and reintegrated back to community life without requiring further medication.

